# Role of expression site switching in the development of resistance to human Trypanosome Lytic Factor-1 in *Trypanosoma brucei brucei*

**DOI:** 10.1016/j.molbiopara.2011.12.004

**Published:** 2012-05

**Authors:** Rudo Kieft, Natalie A. Stephens, Paul Capewell, Annette MacLeod, Stephen L. Hajduk

**Affiliations:** a Department of Biochemistry and Molecular Biology, University of Georgia, Athens, GA 30602, USA; b Wellcome Trust Centre for Molecular Parasitology, Institute of Biodiversity Animal Health and Comparative Medicine College of Medical, Veterinary and Life Sciences, University of Glasgow, Glasgow G61 1QH, UK

**Keywords:** BES, bloodstream expression site, ESAG, expression site associated gene, VSG, variant surface glycoprotein, TLF, Trypanosome Lytic Factor, Hygro, hygromycin, MITat, Molteno institute trypanosome antigen type, HpHbR, haptoglobin hemoglobin receptor, ApoA-1, apolipoprotein A-1, ApoL-1, apolipoprotein L-1, Hpr, haptoglobin related protein, SRA, serum resistance associated, Innate immunity, High density lipoprotein, Human serum resistance, Trypanosome Lytic Factor, Haptoglobin/hemoglobin receptor, Expression site, Variant surface glycoprotein

## Abstract

Human high-density lipoproteins (HDLs) play an important role in human innate immunity to infection by African trypanosomes with a minor subclass, Trypanosome Lytic Factor-1 (TLF-1), displaying highly selective cytotoxicity to the veterinary pathogen *Trypanosoma brucei brucei* but not against the human sleeping sickness pathogens *Trypanosoma brucei gambiense* or *Trypanosoma brucei rhodesiense*. *T. b. rhodesiense* has evolved the serum resistance associated protein (SRA) that binds and confers resistance to TLF-1 while *T. b. gambiense* lacks the gene for SRA indicating that these parasites have diverse mechanisms of resistance to TLF-1. Recently, we have shown that *T. b. gambiense* (group 1) resistance to TLF-1 correlated with the loss of the haptoglobin/hemoglobin receptor (HpHbR) expression, the protein responsible for high affinity binding and uptake of TLF-1. In the course of these studies we also examined TLF-1 resistant *T. b. brucei* cell lines, generated by long-term *in vitro* selection. We found that changes in TLF-1 susceptibility in *T. b. brucei* correlated with changes in variant surface glycoprotein (VSG) expression in addition to reduced TLF-1 binding and uptake. To determine whether the expressed VSG or expression site associated genes (ESAGs) contribute to TLF-1 resistance we prepared a TLF-1 resistant *T. b. brucei* with a selectable marker in a silent bloodstream expression site (BES). Drug treatment allowed rapid selection of trypanosomes that activated the tagged BES. These studies show that TLF-1 resistance in *T. b. brucei* is largely independent of the expressed VSG or ESAGs further supporting the central role of HpHbR expression in TLF-1 susceptibility in these cells.

## Graphical abstract

**Graphical abstract:**

Resistance to Trypanosome Lytic Factor-1 is largely independent of the expressed variant surface glycoprotein (VSG) gene.

**Figure:**
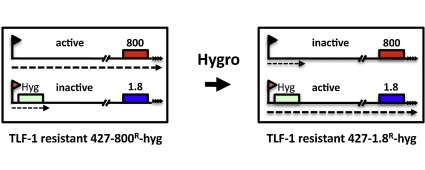

**Highlight:**

► Resistance to TLF-1 killing correlates with the loss of HpHbR expression. ► Changes in VSG expression were found to correlate with TLF-1 susceptibility. ► TLF-1 resistance in *T. b. brucei* is largely independent of the expressed VSG or ESAGs.

## 1 Introduction

Infection and pathogenesis of mammals by African trypanosomes is influenced by innate immune molecules present in the blood of primates. Initially described as a minor subclass of human high density lipoprotein (HDL), Trypanosome Lytic Factor-1 (TLF-1) [1,2] contains apolipoprotein A-1 (apoA-1) and two primate specific proteins apolipoprotein L-1 (ApoL-1) and haptoglobin related protein (Hpr) [3–7]. In addition, high specific activity killing by TLF-1 also requires Hpr bound hemoglobin (Hb)[8]. This HDL subclass is highly toxic to the veterinary pathogen *Trypanosoma brucei brucei*. However, TLF-1 has no activity against the human sleeping sickness parasites *T. b. gambiense* or *T. b. rhodesiense*. The cellular pathway for TLF-1 killing of *T. b. brucei* is now well established. TLF-1 binds to a high affinity haptoglobin hemoglobin receptor (HpHbR) that recognizes Hpr/Hb within the TLF-1 particle and allows endocytosis and lysosomal trafficking [9–12]. *T. b. brucei* can be spared from TLF-1 killing by competition for receptor binding, inhibition of trafficking through the endocytic pathway or by treatment with compounds that elevate lysosomal pH [9,11].

A second trypanolytic serum complex has been identified, TLF-2, which contains ApoA-1 and Hpr as well as IgM [13]. ApoL-1 was not initially detected in TLF-2 by N-terminal sequencing but recent studies support the presence of this apolipoprotein in TLF-2 [14]. In addition to the presence of IgM in TLF-2, another distinguishing feature of the two serum complexes is that TLF-2 is largely devoid of lipid. Aside from the shared apolipoproteins the relationship of these two human defense complexes is largely unknown. However, it is likely that both TLF-1 and TLF-2 play significant roles in the innate immunity that humans have against *T. b. brucei* infection [13].

Within the circulation of primates, TLF-1 and TLF-2 have acted as selective agents leading to the emergence of resistant trypanosomes that cause human disease. This selection resulted in diverse mechanisms of resistance to TLF-1. Beginning with the work of DeGreef and Hamers, it was shown that human serum resistant *T. b. rhodesiense* expressed a novel protein called the serum resistance associated protein (SRA) [15]. The predicted structure of SRA showed that it is a member of the variant surface glycoprotein (VSG) family containing an internal deletion and a unique apoL-1 binding domain [4,16]. Expression and co-localization of SRA within endocytic compartments of *T. b. rhodesiense* have been proposed to allow formation of a TLF-1/SRA binary complex leading to inhibition of trypanosome lysis [4,10,12,17]. It is likely that a gain of function mutation, to an existing VSG gene, gave rise to SRA in an ancestral *T. b. brucei* and was sufficient to confer human infectivity.

Both group 1 and 2 *T. b. gambiense* lack the *SRA* gene and therefore evolved SRA independent mechanism(s) to avoid TLF-1 killing. The mechanism of TLF-1 resistance in *T. b. gambiense* (group 1) is the loss of TLF-1 binding and uptake [18]. We found low-level expression of the HpHbR in seven different field isolates of *T. b. gambiense* relative to the levels found in *T. b. brucei* and *T. b. rhodesiense*. Furthermore, expression of the *T. b. gambiense HpHbR* in receptor deficient *T. b. brucei*, failed to restore TLF-1 binding suggesting that changes to the coding sequence of the *T. b. gambiense* HpHbR may also contribute to reduced TLF-1 binding and killing of *T. b. gambiense* (group 1). The mechanism of TLF-1 resistance in *T. b. gambiense* (group 2) is unknown but does not correlate with loss of receptor-mediated uptake of TLF-1 suggesting a second, SRA-independent mechanism [19].

To study the evolution of TLF-1 resistance in African trypanosomes we reasoned that *in vitro* selection of *T. b. brucei*, by continuous cultivation in the presence of low concentrations of TLF-1, would lead to resistance and might provide insight into the events that led to TLF-1 resistance in the human sleeping sickness parasites. *T. b. brucei* 427-221 was treated with progressively higher concentrations of TLF-1 and over a nine month period parasites with differing levels of resistance to TLF-1 were identified, cloned and characterized [20]. Two striking traits were seen in the highly resistant *T. b. brucei* 427-800 cells. The first was a dramatic reduction in TLF-1 binding and uptake; the second was a periodic change in the expressed VSG in the increasing resistant cells [20]. Transcriptome analysis was consistent with activation of different BESs during the long-term selection. In subsequent studies, we found that HpHbR expression was reduced in TLF-1 resistant *T. b. brucei* 427-800^R^, and other TLF-1 resistant lines, consistent with the loss of TLF-1 uptake [18]. However, the apparent correlation with VSG switching could not be excluded as contributing to the TLF-1 resistance phenotype of these cell lines. Here we present studies that address the role of the expressed VSG and ESAGs in TLF-1 resistance. In order to select for rapid BES switching we introduced the hygromycin resistance gene into an inactive expression site in TLF-1 resistant *T. b. brucei* 427-800^R^. Treatment of *T. b. brucei* 427-800^R^ with hygromycin selected for cells that had switched to the newly activated BES. Using this tagged cell line we are able to show that susceptibility to TLF-1 is largely uninfluenced by the newly expressed VSG or ESAGs. These findings support our proposal that acquired resistance, in TLF-1 resistant *T. b. brucei*, was a consequence of the loss of the HpHbR expression.

## 2 Materials and methods

### 2.1 *In vitro* growth, generation and transfection of *T. b. brucei* cell lines

Bloodstream form *T. b. brucei* Lister 427 (MiTat 1.2) were used in these studies. TLF-1 resistant *T. b. brucei* 427-800^R^ cells were described previously [18]. Prolonged culturing in the absence of TLF-1 resulted in subpopulations of TLF-1 sensitive and resistant cells. Prior to subsequent experiments, cells were cloned by limiting dilution. Transfections were performed using the Amaxa electroporation system (Human T Cell Nucleofactor Kit, program X-001).

### 2.2 SDS-PAGE and northern blot analysis

Total cell protein from 2 × 10^6^ trypanosomes was run on 10% SDS-PAGE and stained with Coomassie Brilliant Blue. For northern blot analysis, radiolabeled probes containing entire open reading frames (ORF) were generated (Prime-It Random Primer Labeling Kit, Stratagene) and hybridized in a 40% (v/v) formamide hybridization mix with the addition of 10% (w/v) dextran sulfate. Final washes were performed with 0.1× SSC, 0.1% SDS at 65 °C for 20 min (1× SSC is 150 mM NaCl, 15 mM sodium citrate, pH 7.4).

### 2.3 TLF-1 purification, labeling, lysis assays and flow cytometry

TLF-1 purification, labeling and lysis assays were performed as described previously [2,8]. Flow cytometry analysis was performed on samples with 3 μg/ml AlexaFluor-488 conjugated TLF-1. Cells were incubated for 1 h at 37 °C, washed 3 times with ice cold 1× PSG (1× PSG is 50 mM NaP_i_, 45 mM NaCl, 55 mM glucose, pH 8.0) and analyzed with a Cyan Cytometer (DAKO).

### 2.4 TLF binding assays

Cells were harvested and resuspended at 3 × 10^7^ cells/ml in pre-chilled HMI9 medium. Prior to the addition of Alexa488 conjugated TLF, cells were pre-incubated for 10 min at 3 °C. Cells were then incubated for 15 min at 3 °C in the presence of 3 μg/ml Alexa488 conjugated TLF and 10 μg/ml hemoglobin. After 3 washes with icecold PBS–glucose (1%), cells were fixed in 1% paraformaldehyde for 15 min on ice and analyzed by immunofluorescence microscopy. Image acquisition was carried out using a Zeiss Axio Observer microscope equipped with an AxioCam HSm Camera and analyzed with AxioVision v4.6 software (http://www.zeiss.com). Images were captured with the same exposure and were contrasted to the same extent.

### 2.5 RT-PCR of the expressed VSGs, ESAGs and HpHbR

Total RNA was isolated with Tripure Isolation Reagent (Roche). cDNA was generated in a Reverse Transcription (RT) reaction (Promega). Control reactions were performed with enolase, as well as reactions without added RT. For cloning and sequencing, PCR products were generated with Platinum High Fidelity Taq Polymerase (Invitrogen), gel purified and cloned into the PCR 2.1 vector (Invitrogen). Both strands were sequenced with M13 forward and reverse primers. VSG and ESAG sequences were compared to the *T. b. brucei* 427 data set (GeneDB).

## 3 Results

### 3.1 VSG switching is not required for gain or loss of TLF-1 susceptibility

**Fig. 1:**
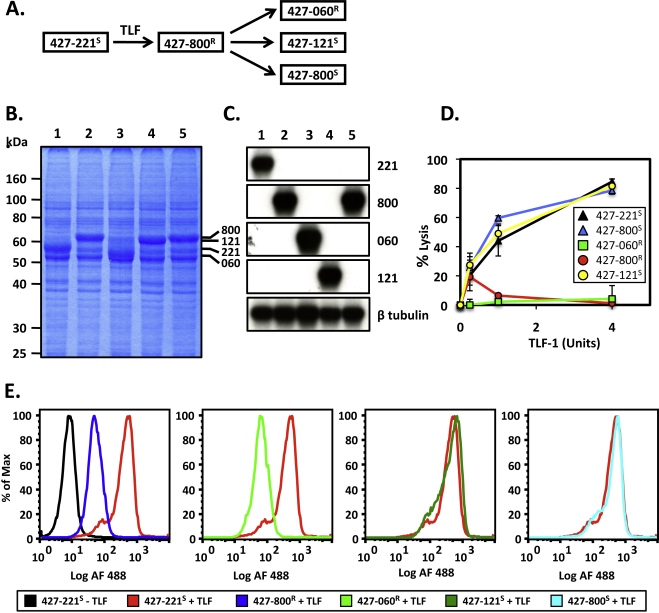
TLF-1 resistance is independent of the expressed VSG and active ES. (A) TLF-1 susceptible and resistant *T. b. brucei* cell lines used in these studies. *T. b. brucei* Lister 427 (MiTat1.2) expressing VSG221, VSG800, VSG060, or VSG121 were defined as TLF-1 resistant (R) or susceptible (S) based on 2 h *in vitro* lysis assays were carried out with increasing concentration of TLF-1 (expressed as units; defined by Hajduk et al., 1989 [2]). (B) SDS-PAGE fractionation of total cell protein from Lane 1, *T. b. brucei* 427-221^S^; Lane 2, *T. b. brucei* 427-800^R^; Lane 3, *T. b. brucei* 427-060^R^; Lane 4, *T. b. brucei* 427-121^S^; Lane 5, *T. b. brucei* 427-800^S^. (C) Northern blot hybridization analysis with specific probes for VSGs and β-tubulin. Lane 1, *T. b. brucei* 427-221^S^; Lane 2, *T. b. brucei* 427-800^R^; Lane 3, *T. b. brucei* 427-060^R^; Lane 4, *T. b. brucei* 427-121^S^; Lane 5, *T. b. brucei* 427-800^S^. β-tubulin was used as a control. (D) *In vitro* susceptibility of the *T. b. brucei* cell lines to TLF. *T. b. brucei* 427-221^S^ (black triangle); *T. b. brucei* 427-800^R^ (red circle); *T. b. brucei* 427-060^R^ (green square); *T. b. brucei* 427-121^S^ (yellow circle); *T. b. brucei* 427-800^S^ (blue triangle). (E) Analysis of Alexa488 conjugated TLF uptake by FAC. Uptake of labeled TLF by *T. b. brucei* 427-221^S^ (red); *T. b. brucei* 427-800^R^ (dark blue); *T. b. brucei* 427-121^S^ (dark green); *T. b. brucei* 427-060^R^ (light green) and *T. b. brucei* 427-800^S^ (light blue). *T. b. brucei* 427-221^S^ in the absence of TLF (black) is shown as a control. In all panels each trypanosome cell line was designated by the expressed VSG and its susceptibility to TLF. (For interpretation of the references to color in this figure legend, the reader is referred to the web version of the article.)

The *T. b. brucei* Lister 427 (MITat1.2) cell line used in these studies is well characterized with respect to its susceptibility to TLF-1 and a complete description of its BESs, VSGs, and ESAGs is available [20,21]. Treatment of TLF-1 sensitive *T. b. brucei* 427-221, expressing the VSG221 (427-221^S^), resulted in the gradual outgrowth of a population of highly resistant parasites that we called *T. b. brucei* 427-800^R^ [20] (Fig. 1A). A feature of *T. b. brucei* 427-800^R^ was the loss of TLF-1 binding and uptake. In addition, SDS-PAGE fractionation of total cellular proteins suggested that the TLF-1 resistant population had switched VSG expression since a major band, migrating at the expected position of the VSG had altered migration (Fig. 1B). Prolonged cultivation of these resistant cells, in the absence of TLF-1, resulted in subpopulations of TLF-1 sensitive and resistant cells. Clonal cell lines were prepared for each of these populations and the VSG expressed in each cell line determined by sequencing of RT-PCR products and by northern blot hybridization using VSG specific probes (Fig. 1C). Each cell line was tested for susceptibility to killing by TLF-1 in a short-term *in vitro* lysis assay (2 h at 37 °C) (Fig. 1D).

Based on the expressed VSG and the susceptibility to TLF-1 the cell lines were designated *T. b. brucei* 427-060^R^, *T. b. brucei* 427-121^S^, and *T. b. brucei* 427-800^S^ (Fig. 1A). We anticipated that resistant cells would show reduced levels of TLF-1 uptake relative to the parental, TLF-1 susceptible *T. b. brucei* 427-221^S^. Incubation of cells with Alexa488 conjugated TLF-1 confirmed that the resistant cells had low levels of TLF-1 uptake. TLF-1 susceptible cells that emerged from long-term growth of *T. b. brucei* 427-800^R^, in the absence of TLF-1, had regained levels of uptake to levels comparable to the parental *T. b. brucei* 427-221^S^ (Fig. 1E). We found that there was considerable diversity in the VSGs expressed by both TLF-1 resistant and susceptible trypanosomes derived from *T. b. brucei* 427-800^R^. When two cell lines that had converted to the TLF-1 susceptible phenotype, were characterized for VSG expression, one was found to express VSG800 while the other expressed VSG121. We also observed that one cell line that retained resistance to TLF-1 expressed VSG060. Based on these finding, it appears that while VSG switching may accompany gain or loss of TLF-1 resistance it is not obligatory, suggesting that susceptibility to TLF-1 is independent of the VSG and perhaps the active BES.

### 3.2 Characterization of the active BES in TLF-1 resistant and susceptible *T. b. brucei*

**Fig. 2:**
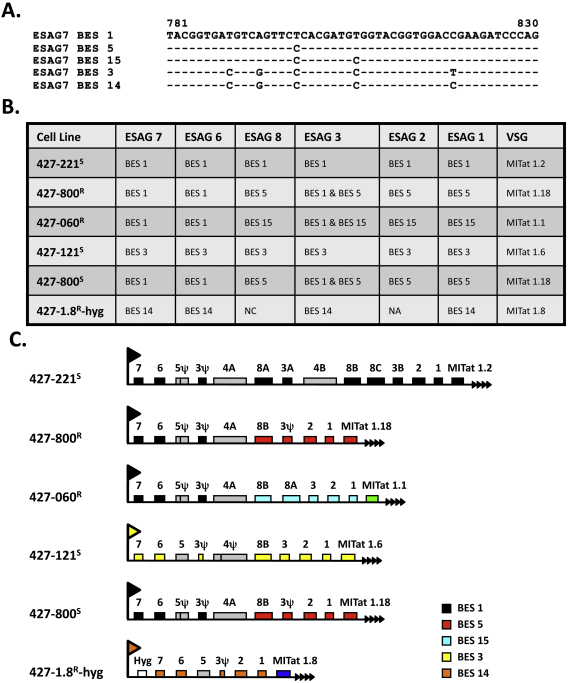
Defining the active ES of the TLF-1 resistant and susceptible T. b. brucei cell lines. (A) Analysis of sequence variation in the transferrin receptor mRNAs. Shown is a portion of the hypervariable sequence for ESAG7 from different BESs. Based on these known sequences we compared RT-PCR sequences for the expressed ESAG7 from the cell lines used in these studies to determine the active expression site. Bloodstream expression site (BES) designations are based on (21). (B) Origin of expressed ESAGs and VSGs. RT-PCR products from ESAG 1, 2, 3, 6, 7, 8 and VSGs were cloned, sequenced and aligned to the *T. brucei* 427 dataset (GeneDB). Sequence polymorphisms from within the genes allow the proper identification of which ESAG is expressed and from which ES it is derived. NA is Not Analyzed; NC is Not Characterized. (C) Summary of the active expression site (ES). RT PCR sequence analysis of the expressed VSG and ESAGs 1, 2, 3, 6, 7 and 8 from the active ES were used to reconstruct the active ES in every cell line. Black boxes are BES 1 derived sequences. Red boxes are BES 5 derived sequences. Turquoise boxes are BES 15 derived sequences. Yellow boxes are BES 3 derived sequences. Orange boxes are BES 14 derived sequences. The green box corresponds to VSG060 (MITat 1.1) and the blue box corresponds with VSG1.8 (MITat 1.8). Grey boxes correspond to genes not taken along in the RT PCR analysis. The white box corresponds to the integrated hygromycin (Hyg) gene. The active ES promoter is determined by which ESAG 7 is expressed. (For interpretation of the references to color in this figure legend, the reader is referred to the web version of the article.)

Since each BES has a characteristic ESAG6 and 7, encoding the heterodimeric transferrin receptor, we sequenced RT-PCR products from the variable region of ESAG7 and compared these sequences with those from known BESs (Fig. 2A) [21]. *T. b. brucei* 427-800^R^ and 427-060^R^ lines express the VSG800 (MITat1.18) and VSG060 (MITat1.1) respectively from the BES1 indicating that VSG060 (MITat1.1) had recombined into the BES1. In contrast, the BES3 which was activated *in situ* in the *T. b. brucei* 427-121^S^ cell line, expresses the MITat1.6 VSG (Fig. 2B and C). The identification of the expressed VSGs and ESAGs allowed us to identify the active BES and approximate the sites of recombination during gene conversion VSG switching events. RT-PCR products from ESAGs 1, 2, 3, 6, 7, 8 and VSGs were cloned, sequenced and aligned to the *T. brucei* 427 dataset (GeneDB). Sequence polymorphisms from within the genes allow the proper identification of which ESAG is expressed and from which BES it is derived. These studies allowed us to evaluate the active BES and ESAGs in the resistant and susceptible cell lines (Table S2). However, because of the extended periods of growth and subsequent cell cloning it was difficult to determine whether the active BES and the expressed ESAGs directly influenced TLF-1 susceptibility.

### 3.3 Selection of BES switching in TLF-1 resistant *T. b. brucei*

**Fig. 3:**
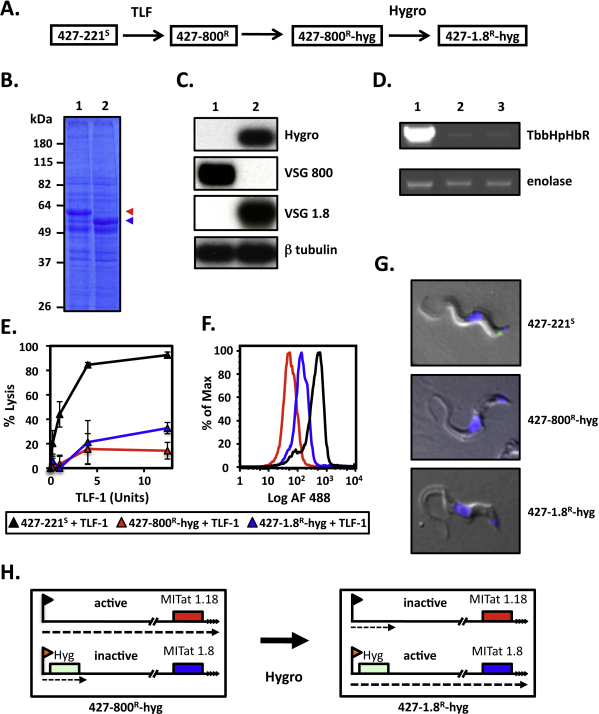
Expression site switching does not correlate with TLF-1 susceptibility. (A) *T. b. brucei* 427-800^R^ were used to introduce a hygromycin resistance gene into the silent BES 14. Treatment with hygromycin (Hygro) selected for cells that had activated BES 14 and now expressed VSG1.8 (MITat 1.8). (B) SDS-PAGE fractionation of total cell protein from *T. b. brucei* 427-800^R^-hyg (lane 1) and *T. b. brucei* 427-1.8^R^-hyg (lane 2). Position of the VSG800 (MITat 1.18; red arrowhead) and VSG1.8 (MITat 1.8; blue arrowhead) are indicated. (C) Northern blot analysis of total RNA from *T. b. brucei* 427-800^R^-hyg (lane 1) and *T. b. brucei* 427-1.8^R^-hyg (lane 2) with specific probes for the hygromycin resistance gene (Hygro), VSG800, VSG1.8 and β-tubulin. (D) Nested RT PCR analysis of TbbHpHbR expression in *T. b. brucei* 427-221^S^ (lane 1), *T. b. brucei* 427-800^R^-hyg (lane 2) and *T. b. brucei* 427-1.8^R^-hyg (lane 3). Enolase expression was analyzed as a loading control in a single round PCR amplification. (E) *In vitro* susceptibility of the *T. b. brucei* cell lines to TLF. *T. b. brucei* 427-221^S^ (black triangle); *T. b. brucei* 427-800^R^-hyg (red triangle); *T. b. brucei* 427-1.8^R^-hyg (blue triangle). (F) Analysis of Alexa488 conjugated TLF uptake by FAC. *T. b. brucei* 427-221^S^ (black); *T. b. brucei* 427-800^R^-hyg (red); *T. b. brucei* 427-1.8^R^-hyg (blue). Note that the Alexa488 fluorescence is plotted on a log scale. (G) Analysis of Alexa488 conjugated TLF binding on live 427-221^S^, 427-800^R^-hyg and 427-1.8^R^-hyg cells at 3 °C. Cells were fixed under non-permeabilizing conditions. (H) Summary of the ES selection using the hygromycin gene inserted into a silent ES. (For interpretation of the references to color in this figure legend, the reader is referred to the web version of the article.)

To circumvent the prolonged time required to allow susceptible cells to emerge from *T. b. brucei* 427-800^R^ the hygromycin resistance gene was introduced at the promoter of the inactive BES14 with the enES2 construct [22] in the TLF-1 resistant *T. b. brucei* 427-800^R^. The resulting cell line, *T. b. brucei* 427-800^R^-hyg, was treated with hygromycin to select cells that had switched to expression of BES14 (Fig. 3A and H). Expression of VSG1.8 (MITat1.8) was verified by SDS-PAGE and northern blot hybridization (Fig. 3B and C). To determine whether the drug induced switching of BES also affected the expression of TbbHpHbR we carried out quantitative RT-PCR analysis using TbbHpHbR specific primers (Fig. 3D and Table S1). The levels of TbbHpHbR mRNA remained low in *T. b. brucei* 427-1.8^R^-hyg cells, consistent with levels observed in the parental *T. b. brucei* 427-800^R^ cells [18].

*In vitro* lysis assays revealed that *T. b. brucei* 427-1.8^R^-hyg and *T. b. brucei* 427-800^R^-hyg were both highly resistant to TLF-1 killing (Fig. 3E). Only at the highest TLF-1 concentrations tested (30 units/ml), did we see that *T. b. brucei* 427-1.8^R^-hyg was slightly more susceptible to TLF-1 killing than *T. b. brucei* 427-800^R^-hyg (∼30% and 10% lysis respectively). In flow cytometry studies, both *T. b. brucei* 427-800^R^-hyg and *T. b. brucei* 427-1.8^R^-hyg showed reduced TLF-1 uptake relative to *T. b. brucei* 427-221^S^ (Fig. 3F). Surprisingly, we saw that *T. b. brucei* 427-1.8^R^-hyg had taken up approximately two-fold more TLF-1 than *T. b. brucei* 427-800^R^-hyg (Fig. 3F). The slight differences in TLF-1 killing seen for these two cell lines was inconsistent with the increased binding and uptake measured by flow cytometry. To determine whether the TLF-1 detected in the *T. b. brucei* 427-1.8^R^-hyg cells by flow cytometry resulted from binding to the flagellar pocket, a prerequisite for receptor-mediated endocytosis in trypanosomes, we carried out low temperature binding studies with TLF-1 (Fig. 3G). Live *T. b. brucei* 427-221^S^, *T. b. brucei* 427-800^R^-hyg and *T. b. brucei* 427-1.8^R^-hyg were incubated for 15 min at 3 °C in the presence of 3 μg/ml Alexa488 conjugated TLF-1, washed and fixed prior to fluorescence microscopy imaging. TLF-1 binding was detectable within the flagellar pocket of the *T. b. brucei* 427-221^S^, but neither of the resistant cells (Fig. 3G). Therefore, although hygromycin induced BES switching resulted in a two-fold increase in cell associated TLF-1, as measured by flow cytometry, there appears to be no detectable binding of TLF-1 to the flagellar pocket in either of the TLF-1 resistant cell lines. It is likely that the slight increase in TLF-1 binding to the *T. b. brucei* 427-1.8^R^-hyg cells may reflect increased, non-specific association of TLF-1 with these cells.

## 4 Discussion

African trypanosomes have evolved diverse mechanisms to evade the cytotoxic activity of human serum thus allowing the parasites to infect humans and ultimately cause disease. Several mechanisms have been described to account for *T. b. rhodesiense* and *T. b. gambiense* resistance to TLF-1 [17–19]. Most *T. b. rhodesiense* isolates express the *SRA* gene and that is sufficient to confer resistance to TLF-1 and normal human serum [23]. Found primarily within endosomal vesicles in *T. b. rhodesiense*, SRA co-localizes with TLF-1 following endocytosis and the two molecules traffic together to the lysosome [12]. Currently it is believed that binding of SRA blocks ApoL-1 insertion into the trypanosome lysosomal membrane thus blocking the formation of a toxic ion channel, however, the precise mechanism is still unknown [4,6].

*T. b. gambiense* (group 1) lacks the *SRA* gene and avoids killing by TLF-1, at least in part, because of decreased receptor-mediated endocytosis of TLF-1. In several isolates of group 1 *T. b. gambiense*, the abundance of HpHbR mRNA is decreased at least 20-fold relative to the levels of the HpHbR mRNA in TLF-1 susceptible *T. b. brucei*. In addition, mutations to the group 1 *T. b. gambiense HpHbR* gene result in amino acid changes that render it incapable of complementing HpHbR deficient *T. b. brucei* [18]. These results suggest that resistance to TLF-1 by group 1 *T. b. gambiense*, is the consequence of multiple events leading to reduced expression of the HpHbR, likely at the level of mRNA stability, and accumulation of point mutations that reduced the affinity of the *T. b. gambiense* HpHbR for TLF-1. In the course on our studies of *T. b. gambiense*, we observed that long-term *in vitro* cultivation of *T. b. brucei*, in the presence of TLF-1, selected for cells that failed to take-up TLF-1 and this correlated with a decrease in HpHbR mRNA expression [18,20].

In this paper, we examined whether the expressed VSG or ESAGs contributed to the acquired resistance of *T. b. brucei* to TLF-1. Prolonged cultivation of *T. b. brucei* 427-800^R^, in the absence of TLF-1 allowed for the outgrowth of both TLF-1 resistant and susceptible clonal cell lines (Fig. 1). This instability of resistance could have resulted from BES switching as described for the loss of *SRA* expression in *T. b. rhodesiense* [17]. On the contrary, we found that active BES and the expressed VSG had little effect on susceptibility of TLF-1 resistant trypanosomes. Further, to directly determine whether BES switching could alter susceptibility to TLF-1, a drug marker was introduced into an inactive BES and cells that had switched to the tagged BES were selected for with hygromycin (Fig. 3). The drug resistant cell line, *T. b. brucei* 427-1.8^R^-hyg, remained highly resistant to TLF-1. Interestingly, while BES switching had little effect on TLF-1 susceptibility we did observe a two-fold increase in TLF-1 binding as measured by flow cytometry (Fig. 3F). However, this binding is not localized to the flagellar pocket, the only site for receptor-mediated endocytosis in trypanosomes. We suggest that the two-fold increase in TLF-1 binding to *T. b. brucei* 427-1.8^R^-hyg is the consequence of low affinity to the MITat1.8 VSG. Since this low level binding would be spread over the entire cell surface it might not be visible by fluorescence microscopy. Taken together these studies argue that the expressed VSG and ESAGs do not play a major role in TLF-1 killing of *T. b. brucei* and provide further support for our hypothesis that decreased expression and mutations to the *HpHbR* are the major determinants in TLF-1 resistance in both TLF-1 resistant *T. b. brucei* and *T. b. gambiense*.

## References

[1] Rifkin M.R. (1978). Identification of the trypanocidal factor in normal human serum: high-density lipoprotein. Proc Natl Acad Sci U S A.

[2] Hajduk S.L., Moore D.R., Vasudevacharya J., Siqueira H., Torri A.F., Tytler E.M. (1989). Lysis of *Trypanosoma brucei* by a toxic subspecies of human high density lipoprotein. J Biol Chem.

[3] Smith A.B., Esko J.D., Hajduk S.L. (1995). Killing of trypanosomes by human haptoglobin related protein. Science.

[4] Vanhamme L., Paturiaux-Hanocq F., Poelvoorde P., Nolan D.P., Lins L., Van Den Abbeele J. (2003). Apolipoprotein L-I is the trypanosome lytic factor of human serum. Nature.

[5] Shiflett A.M., Bishop J.R., Pahwa A., Hajduk S.L. (2005). Human high density lipoproteins are platforms for the assembly of multi-component innate immune complexes. J Biol Chem.

[6] Perez-Morga D., Vanhollebeke B., Paturiaux-Hanocq F., Nolan D.P., Lins L., Homble F. (2005). Apolipoprotein L-I promotes trypanosome lysis by forming pores in lysosomal membranes. Science.

[7] Molina-Portela M.P., Samanovic M., Raper J. (2008). Distinct roles of apolipoprotein components within the trypanosome lytic factor complex revealed in a novel transgenic mouse model. J Exp Med.

[8] Widener J., Nielsen M.J., Shiflett A., Moestrup S., Hajduk S. (2007). Hemoglobin is a co-factor of human trypanosome lytic factor. PLoS Pathogens.

[9] Hager K.M., Pierce M.A., Moore D.R., Tytler E.M., Esko J.D., Hajduk SL (1994). Endocytosis of a cytotoxic human high density lipoprotein results in disruption of acidic intracellular vesicles and subsequent killing of African trypanosomes. J Cell Biol.

[10] Oli M.W., Cotlin L.F., Shiflett A.M., Hajduk S.L. (2006). Serum resistance associated protein blocks lysosomal targeting of trypanosome lytic factor in *Trypanosoma brucei*. Eukaryot Cell.

[11] Vanhollebeke B., De Muylder G., Nielsen M.J., Pays A., Tebabi P., Dieu M. (2008). A haptoglobin-hemoglobin receptor conveys innate immunity to *Trypanosoma brucei* in humans. Science.

[12] Stephens N.A., Hajduk S.L. (2011). Endosomal Localization of the serum resistance-associated protein in African trypanosomes confers human infectivity. Eukaryot Cell.

[13] Raper J., Fung R., Ghiso J., Nussenzweig V., Tomlinson S. (1999). Characterization of a novel trypanosome lytic factor in human serum. Infect Immun.

[14] Bullard W., Kieft R., Capewell P., Veitch N.J., Macleod A., Hajduk S.L. (2012). Haptoglobin-hemoglobin receptor independent killing of African trypanosomes by human serum and trypanosome lytic factors. Virulence.

[15] DeGreef C., Hamers R. (1994). The serum resistance-associated (SRA) gene of *Trypanosoma brucei rhodesiense* encodes a variant surface glycoprotein-like protein. Mol Biochem Parasitol.

[16] Campillo N., Carrington M (2003). The origin of the serum resistance associated (SRA) gene and a model of the structure of the SRA polypeptide from *Trypanosoma brucei rhodesiense*. Mol Biochem Parasitol.

[17] Xong H.V., Vanhamme L., Chamekh C.E., Chimfwembe, Van Den Abbeele J., Pays A. (1998). A VSG expression site-associated gene confers resistance to human serum in *Trypanosoma rhodesiense*. Cell.

[18] Kieft R., Capewell P., Turner C.M., Veitch N.J., MacLeod A., Hajduk S. (2010). Mechanism of *Trypanosoma brucei gambiense* (group 1) resistance to human trypanosome lytic factor. Proc Natl Acad Sci U S A.

[19] Capewell P., Veitch N.J., Turner C.M.R., Raper J., Berriman M., Hajduk S.L. (2011). Differences between *Trypanosoma brucei gambiense* Groups 1 and 2 in their resistance to killing by trypanolytic factor 1. PLoS Neg Trop Dis.

[20] Faulkner S.D., Oli M.W., Kieft R., Cotlin L., Widener J., Shiflett A. (2006). In vitro generation of human high density lipoprotein resistant *Trypanosoma brucei brucei*. Eukaryot Cell.

[21] Hertz-Fowler C., Figueiredo L.M., Quail M.A., Becker M., Jackson A., Bason N. (2008). Telomeric expression sites are highly conserved in *Trypanosoma brucei*. PLoS One.

[22] Rudenko G., Blundell P.A., Taylor M.C., Kieft R., Borst P. (1994). VSG gene expression site control in insect form *Trypanosoma brucei*. EMBO J.

[23] Turner C.M.R., McLellan S., Lindergard L.A.G., Bisoni L., Tait A., MacLeod A. (2004). Human infectivity trait in *Trypanosoma brucei*: stability, heritability and relation to SRA expression. Parasitology.

