# Supplementary material for: Role of expression site switching in the development of resistance to human Trypanosome Lytic Factor-1 in Trypanosoma brucei brucei
Source: Mol Biochem Parasitol. 2012 May;183(1):8–14. doi: 10.1016/j.molbiopara.2011.12.004 (PMC3343262; doi:10.1016/j.molbiopara.2011.12.004)
Supplement: Supplementary file 1 [file mmc1.zip › mmc1.docx]

**Supplementary Data:**

**Table 1.** *Oligonucleotides used in (RT) PCR experiments*

| Name | Orientation | Sequence |
| --- | --- | --- |
| Oligo dT | antisense | 5’-GCTCTAGATTTTTTTTTTTTTTTTTTTT-3’ |
| Splice leader RNA | sense | 5’-CCGGAATTCGCTATTATTAGAACAGTTTCT-3’ |
| VSG 3’ Xba I conserved | antisense | 5’-cccgcctctagacgtgttaaaatatatcag-3’ |
| ESAG 1 | antisense | 5’-ATCAAARGATGCAATGAGGG-3’ |
| ESAG 2 | antisense | 5’-ATATTTATCCTGTTCCAAATTCAC-3’ |
| ESAG 3 | antisense | 5’-AATAGGCTGTCCGGGAAAAT-3’ |
| ESAG 8 | antisense | 5’-TCAAAATATATGCACACCTCG-3’ |
| ESAG 6/7 | antisense | 5’-gctctagacatcactgcattttttgcttc-3’ |
| enolase | antisense | 5’-ccaaccgggaaagccaaatttagc- 3’ |

Restriction sites used for cloning are underlined.
