# Supplementary material for: Role of expression site switching in the development of resistance to human Trypanosome Lytic Factor-1 in Trypanosoma brucei brucei
Source: Mol Biochem Parasitol. 2012 May;183(1):8–14. doi: 10.1016/j.molbiopara.2011.12.004 (PMC3343262; doi:10.1016/j.molbiopara.2011.12.004)
Supplement: Supplementary file 2 [file mmc2.zip › mmc2.docx]

**Supplementary Data:**

**Table 2.** *Expressed ESAG’s from used cell lines*

| Cell line | ESAG 7 | ESAG 6 | ESAG 8 | ESAG 3 | ESAG 2 | ESAG 1 |
| --- | --- | --- | --- | --- | --- | --- |
| 427-221^S^ | Tb427.TAR40.2 | Tb427.TAR40.3 | Tb427.TAR40.14  Tb427.TAR40.15 | Tb427.TAR40.5  Tb427.TAR40.10  Tb427.TAR40.16 | Tb427.TAR40.18 | Tb427.TAR40.20 |
| 427-800^R^ | Tb427.TAR40.2 | Tb427.TAR40.3 | Tb427.TAR98.7 | Tb427.TAR40.5  Tb427.TAR98.8 | Tb427.TAR98.9 | Tb427.TAR98.11 |
| 427-060^R^ | Tb427.TAR40.2 | Tb427.TAR40.3 | Tb427.TAR126.7  Tb427.TAR126.8 | Tb427.TAR40.5  Tb427.TAR126.9 | Tb427.TAR126.10 | Tb427.TAR126.12 |
| 427-121^S^ | Tb427.TAR15.2 | Tb427.TAR15.3 | Tb427.TAR15.7 | Tb427.TAR15.5 | Tb427.TAR15.9 | Tb427.TAR15.11 |
| 427-800^S^ | Tb427.TAR40.2 | Tb427.TAR40.3 | Tb427.TAR98.7 | Tb427.TAR40.5  Tb427.TAR98.8 | Tb427.TAR98.9 | Tb427.TAR98.11 |
| 427-1.8^R^-hyg | Tb427.TAR10.1 | Tb427.TAR10.2 | NC | Tb427.TAR10.4 | NA | Tb427.TAR10.7 |

Sequences are aligned with TAR clones representing the Lister 427 BES’s (Hertz-Fowler *et al.,* 2008)

NC is Non Characterized; NA is Not Analyzed
